# The folding, stability and function of lactose permease differ in their dependence on bilayer lipid composition

**DOI:** 10.1038/s41598-017-13290-7

**Published:** 2017-10-12

**Authors:** Heather E. Findlay, Paula J. Booth

**Affiliations:** Department of Chemistry, Kings College London, Britannia House, 7 Trinity Street, London, SE1 1DB UK

## Abstract

Lipids play key roles in Biology. Mechanical properties of the lipid bilayer influence their neighbouring membrane proteins, however it is unknown whether different membrane protein properties have the same dependence on membrane mechanics, or whether mechanics are tuned to specific protein processes of the protein. We study the influence of lipid lateral pressure and electrostatic effects on the *in vitro* reconstitution, folding, stability and function of a representative of the ubiquitous major facilitator transporter superfamily, lactose permease. Increasing the outward chain lateral pressure in the bilayer, through addition of lamellar phosphatidylethanolamine lipids, lowers lactose permease folding and reconstitution yields but stabilises the folded state. The presence of phosphatidylethanolamine is however required for correct folding and function. An increase in headgroup negative charge through the addition of phosphatidylglycerol lipids favours protein reconstitution but is detrimental to topology and function. Overall the *in vitro* folding, reconstitution, topology, stability and function of lactose permease are found to have different dependences on bilayer composition. A regime of lipid composition is found where all properties are favoured, even if suboptimal. This lays ground rules for rational control of membrane proteins in nanotechnology and synthetic biology by manipulating global bilayer properties to tune membrane protein behaviour.

## Introduction

The interaction of proteins and lipids in membranes is central to cellular function. Whilst proteins are key players in regulating transport and communication across membranes, the lipids are not just passive bystanders that provide an impermeable layer, but are also active participants in membrane reactions^1,2^. Certain lipids, such as inositol phospholipids, operate in terms of chemically specificity^3^, whilst others interact directly with membrane proteins as in the case of cardiolipin with cytochrome oxidase^4,5^. Annular lipids, which are proposed to surround proteins in membranes, have also attracted attention^6,7^. However, there is another, relatively unexplored lipid mechanism, in which global properties of the lipid bilayer regulate protein behavior. The mechanical and charge properties of the bilayer affect membrane protein folding, stability, topology and function^8–13^.

Many different lipids are found in biology, providing a means to maintain the fluid bilayer that is crucial to membrane integrity and function. Manipulating lipid compositions *in vitro* proffers the chance to probe the influence of such global bilayer properties on membrane proteins. A range of distinct but interdependent global properties are involved; including stored curvature elastic stress, lipid monolayer curvature, lipid lateral pressure, bending rigidity, area expansion modulus, hydrophobic thickness and headgroup charge^14,15^.

Phosphatidylcholine (PC) lipids (e.g. L-α-1,2-dioleoylphosphatidylcholine, DOPC) form monolayers with relatively flat interface with water that tend to form fluid, lamellar (bilayer) phases. In contrast, phosphatidylethanolamine (PE) lipids like L-α-1,2-dioleoylphosphatidylethanolamine (DOPE) have very different properties. The ethanolamine headgroup contains a primary amine that can form both intra- and inter-lipid hydrogen bonds, which increases the transition temperatures and significantly alters the hydration at the water:lipid interface. The intermolecular forces and reduced size of the headgroup compared with PC lipids also contributes to a greater desire to curve towards an aqueous phase and form inverted hexagonal (non-lamellar) phases^16–18^. Addition of DOPE to a DOPC bilayer increases the monolayer curvature and the stored curvature elastic stress of the PC/PE bilayer. An effect of introducing PE lipids into a PC bilayer is to redistribute the lateral pressure; resulting in a decrease in the headgroup region and an increase in the chain region^19^. This alteration of lateral pressure by PE and the proposed resultant effects on proteins has previously been discussed^20–22^. Anionic lipids are also a major component of biological membranes with headgroups such as phosphatidylserine (PS) and phosphatidylglycerol (PG) common. Similarly to DOPC, 1,2-dioleoyl-glycero-3-phosphoglycerol (DOPG) forms a fluid lamellar bilayer in physiological conditions, and the addition of DOPE increases the monolayer curvature^23^ with predicted corresponding effects on the lateral pressure profile.

Correct folding is an essential process for any protein. The folding feat has been extensively explored for water soluble proteins, but there is considerable less known for membrane proteins, especially in lipid membranes. It has previously shown that lipid composition affects protein folding, topology and function and we have postulated that different membrane protein properties, such as insertion, folding, stability and function are likely to be favoured by different membrane lateral pressures^24–26^. Increasing the amount of PE lipids in a PC bilayer has been found to increase the stability of the KcsA potassium channel^11^ and activation energy for insertion of a transmembrane helix have been found to increase with increasing PE^27^. This increase in activation energy was assigned to the difficulty of inserting a helix across the bilayer when the pressure in the lipid chain region is higher. Similarly increasing the PE content of bilayers decreased the folding yield of bacteriorhodopsin, which was inserted into PC/PE bilayers from a partially denatured state^28^. The results pointed to the increased chain pressure hindering insertion of a folding protein across the bilayer. These studies however are on model peptides/proteins, with bacteriorhodopsin being an unusually stable protein, and the results on lipid effects on folding have not been generalised in any systematic manner.

In parallel to our studies on lipid influence of membrane protein folding, we have also been advancing mechanistic folding work to families of larger, flexible membrane proteins, including the vast ubiquitous major facilitator superfamily (MFS) of transporters. The transporters are relatively large with predominantly helical, dynamic structures that are usually arranged in, two, 6-helical domains. We have shown that the MFS galactose and lactose transporters (GalP and LacY) can be folded from a partially denatured state in urea into lipid bilayer vesicles^29,30^. Moreover, we found a dependence of folding yield for the MFS galactose transporter, GalP, where the folding yield increased with PE to give optimal recovery of secondary structure with 0.6 DOPE (mole fraction) present in DOPE/DOPC lipid vesicles. Higher PE content reduced the yield of folded protein^29^.

Lipid composition also influences the transport activity and topology of the MFS lactose transporter LacY *in vitro* and *in vivo* ^31,32^. In the absence of the dominant *E. coli* membrane lipid, PE, the N domain of LacY is flipped into an inverse topology, upside down in the membrane compared to the C domain^33–36^. This has been assigned primarily to a charge effect across the membrane. The transport activity of LacY has been shown to be affected by the surrounding lipid composition both in modified *E. coli* strains *in vivo* and *in vitro* reconstituted protein^31,32^. Previous folding work using chemical denaturants in a detergent micelle system has demonstrated that LacY can be reversibly folded in a similar manner and with a similar free energy of folding as GalP^30^. Additionally, there are 3D structures available of LacY in a variety of conformations^37,38^. Together this suggests that LacY is an excellent candidate protein for a more systematic study of lipid-membrane protein interactions.

Here, we test our hypotheses that lipid bilayer properties have different effects on different membrane protein properties, and begin to generalise previous results on the influence of lateral pressure on membrane proteins to an archetypal member of one of the largest transporter families. We investigate the influence of DOPE and 1,2-dioleoyl-glycero-3-phosphoglycerol (DOPG) on LacY folding, stability and function. Specifically we address: a) the efficiency of reconstitution into lipid bilayers, of LacY purified from native membranes as a folded state in detergent micelles, b) the stability of the reconstituted LacY, c) the domain topology of LacY reconstituted into bilayers, d) the transport function of reconstituted LacY and e) the folding yield of LacY, folded from a urea denatured state into bilayers. In all cases, we use a three component lipid system based on a non-native lipid DOPC to provide a basic bilayer. DOPG is incorporated as this is a native *E. coli* lipid that forms bilayers but introduces negative charge into the headgroup region. Additionally we add DOPE, which is the dominant headgroup in LacY’s native *E. coli* membranes. We explore the full range of compositions of this 3 component DOPC/DOPG/DOPE lipid system to give a comprehensive picture of the dependence of different membrane protein parameters that are critical to function, using DOPE content as a proxy for chain lateral pressure and DOPG content for negative charge.

## Results

**Figure 1:**
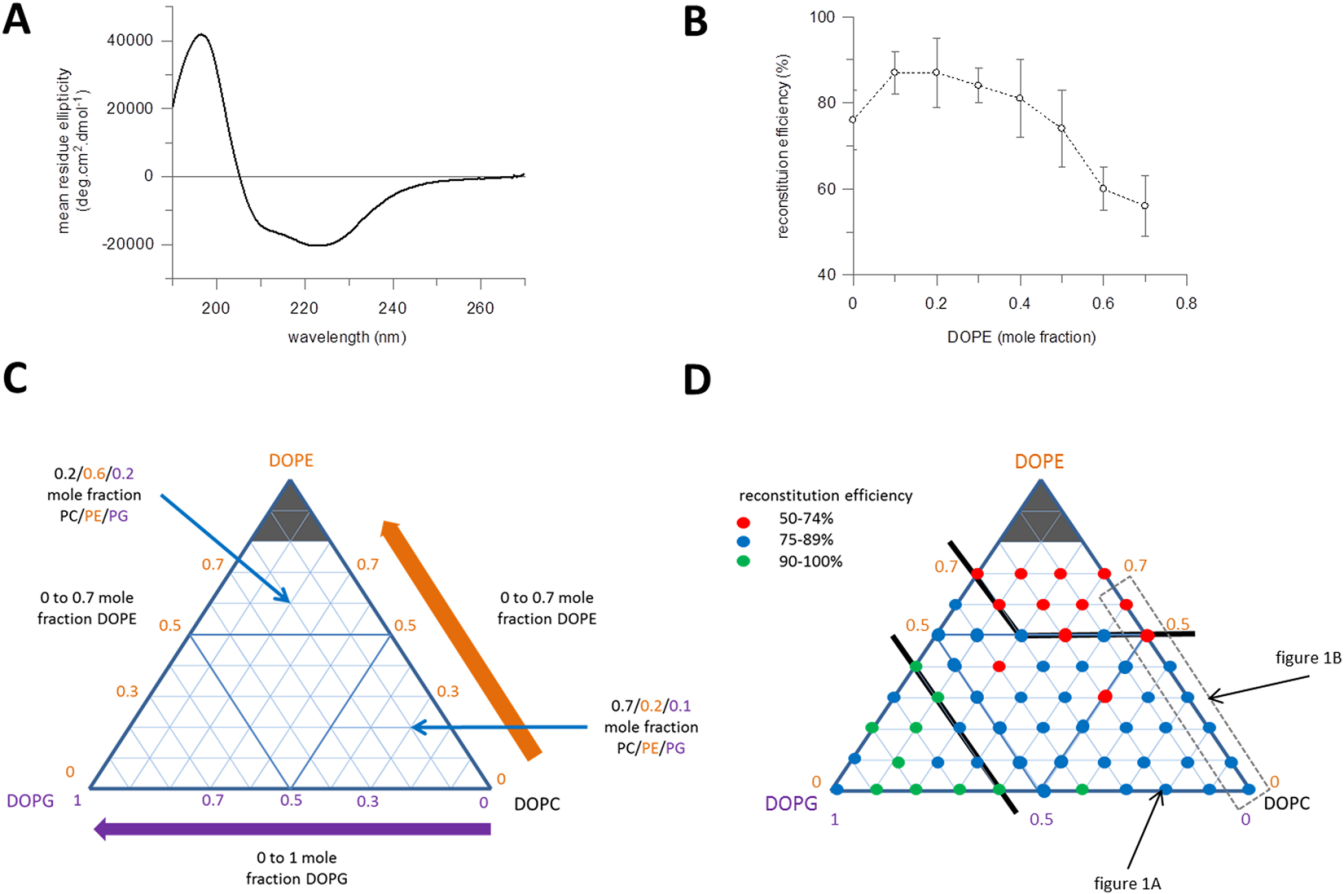
Reconstitution of LacY in lipid vesicles with different lipid compositions. (**A**) Synchrotron CD spectrum of LacY reconstituted into 0.8/0.2 DOPC/DOPG (mole fraction). (**B**) Reconstitution of LacY in vesicles composed of DOPC and DOPE mixtures (n = 5, error bars are ±SD). The concentration of reconstituted protein in each sample was measured by Markwell-Lowry assay, from this the reconstitution efficiency was calculated as a percentage of total protein originally added. DOPE concentration given as mole fraction in DOPE/DOPC vesicles. Increasing concentration of non-lamellar lipids results in decreasing reconstitution efficiency. (**C**) Diagrammatic representation of the tertiary lipid mixes used throughout the study. Axes represent mole fraction of the three lipids. Using DOPC as the base lipid, the axis along the base of the triangle increases right to left from 0 to 1 mole fraction of DOPG (purple). The two other triangle axes increase from 0 to 0.7 mole fraction DOPE (orange, from either DOPG only or DOPC only, with the latter right hand triangle edge representing the DOPC/DOPE compositions in Fig. 1B). Thus the edges of the triangle are two component mixtures, for example of DOPC and DOPG along the baseline axis. Compositions within the triangle are all tertiary mixtures. Two example tertiary mixes – 0.2/0.6/0.2 PC/PE/PG and 0.7/0.2/0.1 PC/PE/PG are highlighted. (**D**) Diagrammatic representation of the reconstitution of LacY in vesicles composed of DOPC, DOPE and DOPG mixtures. Coloured dots show the reconstitution efficiency at different lipid compositions; red dots indicate the lowest efficiency with 50–74% of the protein being successfully reconstituted, blue dots indicate efficiencies of 75–89% and green dots the highest reconstitution efficiencies of 90–100%. The lipid compositions corresponding to the individual spectrum in 1 A and binary mixtures in 1B have been indicated with arrows and a dashed box. The solid black lines are added as a guide to identify the lipid composition regions that gives rise to the differing efficiency regimes. LacY reconstitutes efficiently in most bilayer conditions. High concentrations of DOPE and DOPG inhibit and enhance reconstitution efficiency respectively.

Liposomes were prepared with mixtures of the neutral lipid DOPC, the anionic lipid DOPG and the non-lamellar lipid DOPE, comprised of a range of 0–1 mole fraction DOPC and DOPG and 0–0.7 mole fraction DOPE in intervals of 0.1. Higher fractions of DOPE were not used as Large Unilamellar Vesicles (LUVs) could not be formed with those compositions. These tertiary mixtures can be visualised as a triangle (e.g. Fig. 1C) where moving in the direction of the DOPG axis point represents an increase in membrane surface charge and moving in the direction of the DOPE axis point corresponds to an increase in bilayer rigidity and lateral pressure within the core of the bilayer. Purified LacY in n-dodecyl-β-D-maltopyranoside (DDM) detergent was reconstituted into these liposomes as previously described^39^. Vesicles were first pre-saturated with the detergent n-octyl-β-D-glucopyranoside (OG), before addition of the protein and incubated to allow insertion into the bilayer. The excess detergent was then removed by multiple exchanges with :Bio-Beads^TM^. It is important that as much detergent as possible is removed to minimise its impact on the bilayer, and the high critical micellar concentration (CMC) of OG facilitates this. The amount of residual detergent can be measured using a colorimetric assay^40^, with a typical result giving a final concentration of approximately 1 detergent molecule per 800–1000 lipid molecules in the bilayer (see Supplementary Figure 3). The reconstitution efficiency, secondary structure, topology, transport and stability of the protein was assessed across the full range of lipid compositions. Protein secondary structure was determined by circular dichroism (CD). The proteoliposomes of reconstituted LacY have a high background absorbance, such that CD spectra obtained in a conventional in-house machine have limited range, down to approximately 215 nm. Therefore in order to obtain full spectra allowing for deconvolution of secondary structure, samples were run using synchrotron radiation circular dichroism (SRCD). SRCD beamlines have a higher light intensity and improved signal/noise through the far UV, which enables the collection of more data from difficult samples, including down to 190 nm in lipids, thus giving a sufficient spectrum to estimate secondary structure content. Thus for example the SRCD spectrum of reconstituted LacY in a binary mixture of DOPC and DOPG of 0.8 and 0.2 mole fractions respectively (0.8/0.2 DOPC/DOPG) (Fig. 1A) gives an α-helical content of 75%, similar to that observed in the 3D crystal structure. The total protein concentration in the liposomes post-reconstitution was measured by the Markwell-Lowry assay^41^ and the reconstitution efficiency calculated as a percentage of originally added protein. LacY has a high reconstitution efficiency of >75% in DOPC/DOPE vesicles with DOPE mole fractions up to 0.5 but as the amount of non-lamellar DOPE is increased above 0.5 mole fraction the efficiency starts to decrease (Fig. 1B). Expanding the analysis across all possible lipid mixes shows that PE is a dominant factor influencing reconstitution efficiency, although high concentration of DOPG did promote reconstitution to between 90–100% (Fig. 1D, coded green). Values for reconstitution efficiency at the full range of lipid compositions studied are given in Supplementary Table 1.

### Stability

**Figure 2:**
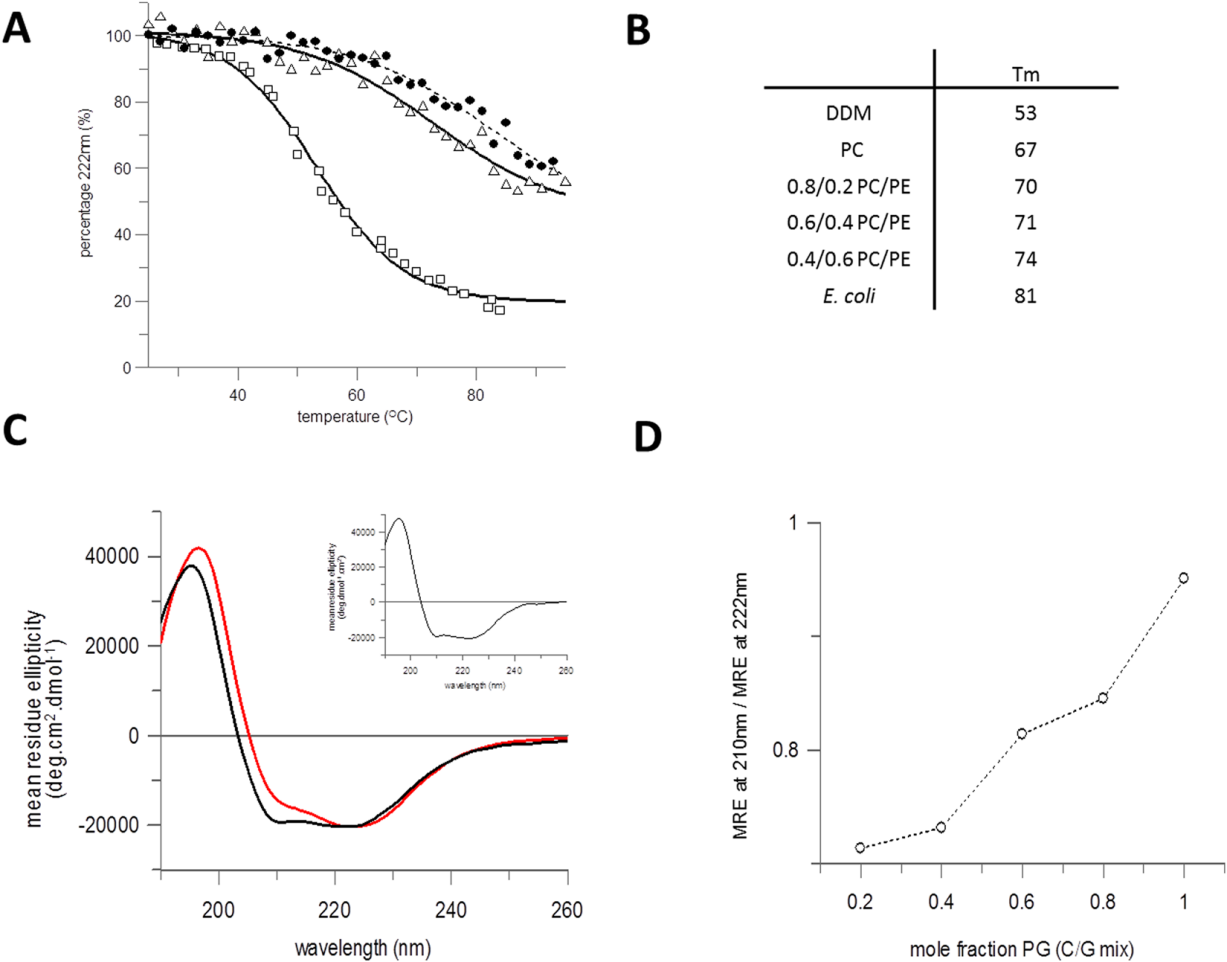
Thermal stability of LacY. (**A**) Thermal denaturation of LacY in DDM detergent micelles (open squares), 0.4/0.6 (mole fraction) DOPC/DOPE vesicles (open triangles) and *E. coli* vesicles (closed circles, dashed line) measured by CD signal at 222 nm. (**B**) Melting temperature midpoints measured by CD thermal denaturation (as in A). (**C**) Synchrotron CD spectra of reconstituted LacY in DOPG only vesicles (solid black line) and 0.8/0.2 mole fraction (solid red line) DOPC/DOPG vesicles. DOPG is concentration given as mole fraction in DOPG/DOPC vesicles. A spectrum of LacY in DDM micelles is shown in an inset for comparison; the DDM spectrum is very similar to the DOPG only spectrum. (**D**) Dependence of the CD spectra on DOPG is shown by comparing the mole fraction DOPG in a binary PC/PG mix with the ratio of the mean residue ellipticity (MRE) at 210 nm to the MRE at 222 nm.

LacY is significantly stabilised when reconstituted into a bilayer environment compared to the detergent micelles the protein used for purification. LacY in DDM micelles is susceptible to denaturation by urea, with far UV SRCD indicating that a third of the secondary structure is lost in 8 M urea (Figure S1A and^30^). In contrast, the protein is stable to urea denaturation in liposomes, with no change in secondary structure occurring in 8M urea (as shown by the spectra for 0.8/0.2 mole ratio DOPC/DOPG in Figure S1B). Thermal stability was assessed by circular dichroism, monitoring the loss of secondary structure during heating. It was not possible to gain enough beamtime for SRCD with appropriate temperature control to obtain full spectra so measurements were made on our specially adapted Aviv commercial machine. Since the complete spectra (characteristic of helical structure) could not be obtained for liposome samples, the signal intensity at 222 nm was monitored with temperature. As LacY is almost entirely helical in structure, the change at 222 nm is a reasonable proxy for loss of secondary structure and comparative stability. The thermal stability was compared for LacY in DDM and reconstituted into different PC/PE/PG lipid mixtures. For mixes of PC and PE, thermal denaturation curves could be fitted to a sigmoidal equation to give a melting midpoint, T_m_, for comparison. LacY in DDM loses almost all of its structure when heated to 80 °C and has a low T_m_ at 53 °C, whereas protein reconstituted into DOPC/DOPE (0.4/0.6) mixtures only loses about half the secondary structure and has a higher T_m_ of 74 °C (Fig. 2A). Increasing the PE content results in a small increase in T_m_ compared to DOPC alone, with the Tm being 67 °C in DOPC, 70 °C with 0.2 mole fraction DOPE present, 71 °C with 0.4 DOPE and 74 °C with 0.6 DOPE present. These values are still somewhat below that observed with protein reconstituted into native *E. coli* lipid extracts, where the T_m_ was 81 °C (Fig. 2A).

LacY reconstituted into bilayers containing DOPG results in changes in the CD spectrum that were not observed in DOPC or DOPE. The spectrum in solely DOPG was more similar to that in DDM micelles, than that in mixes of DOPC/DOPG with mole fractions of DOPG < 0.2; in particular a different 210/222 nm band intensity ratio was observed for DOPG alone versus DOPC/DOPG (Fig. 2C,D). These differences in far UV CD spectra for helical proteins in lipids have been correlated with the differing hydrophobic environment of transmembrane versus soluble domains^42^. The change in LacY fold at high DOPG could suggest a more exposed structure. Therefore monitoring the 222 nm band is no longer a good indication of purely helical reduction when DOPG is present, and although a large reduction in the intensity of this band occurred upon heating in DOPC/DOPG liposomes it did not fit to a simple sigmoidal equation (Figure S1C).

### Topology

**Figure 3:**
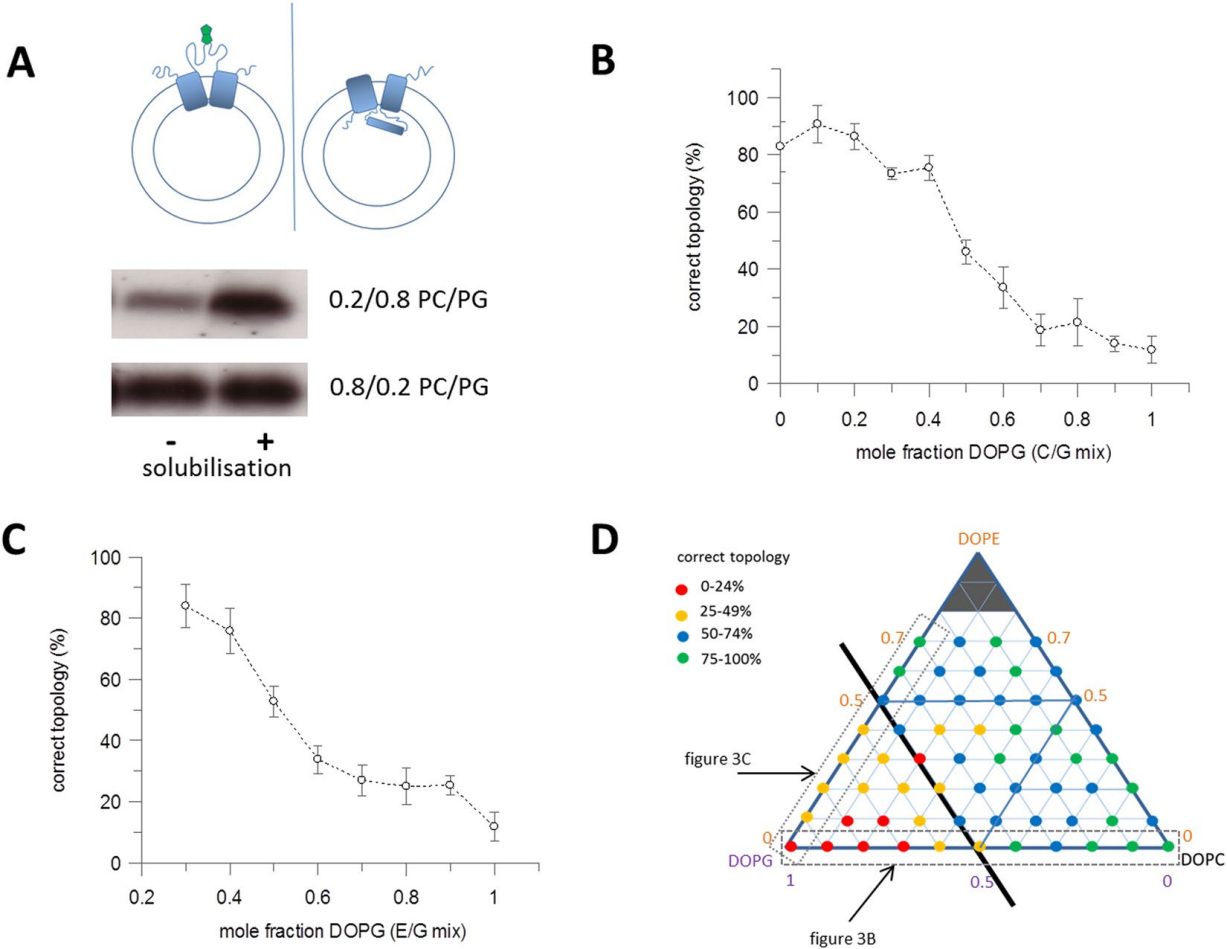
LacY topology changes in lipid vesicles with different lipid composition. (**A**) LacY with a single-cysteine (S146C) in the long interdomain loop was reconstituted into lipid vesicles. Schematic shows the 2 LacY domains in blue, with the correct topology on the left (biotin label attached to S164C shown in green) and inverted N domain topology with no labelling on the right. The amount of biotin labelling of S146C was compared before and after solubilisation of the vesicles by OG detergent. Equal amounts of labelling indicated correct topology. Increasing amounts of an incorrect topology present resulted in a decrease in the extent of labelling prior to OG solubilisation. Labelling was quantified from SDS-PAGE gels, as shown, with fluorescent avidin or using a fluorescence-based biotinylation assay. The two lanes labelled “−“ and “+” indicate before and after solubilisation of vesicles by OG. (**B**) Dependence of correct topology of reconstituted LacY on DOPC/DOPG composition (n = 5, error bars ± SD). DOPG concentration is given as mole fraction in DOPC/DOPG vesicles. (**C**) Correct topology of LacY reconstituted into bilayers composed of DOPE and DOPG (n = 5, ±SD). (**D**) Diagrammatic representation of the structural changes in different lipid compositions. Red dots represent the lowest percentage of protein in the correct topology, from 0–24%, yellow dots from 25–49%, blue dots from 50–74% and green dots the highest percentage, from 75–100%. Grey dashed and dotted boxes highlight the two component lipid mixes shown in Fig. 3B,C respectively. Bilayers with high percentages of DOPG show an increase in inverted topology.

LacY is known to form structures with an inverted N-terminal domain *in vitro* in liposomes lacking PE or containing PG or cardiolipin, where the first six transmembrane helices have a reversed orientation and the seventh helix is expelled entirely from the bilayer. Despite this structural rearrangement, the partially inverted LacY is still capable of facilitating transport across the bilayer^43^. The topology of reconstituted LacY was determined with a single cysteine mutant, S146C, in which the wild-type cysteine residues were replaced by various small aliphatic residues that allow the retention of activity as previously described in^44^. The single Cys is introduced at position 146 in the long loop between the N- and C-terminal domains. In the correct domain topology this loop is exposed on the outside of the liposomes and can be labelled, whilst if the N-domain is inverted before site 146 it is inside the liposome and cannot be labelled by a membrane impermeable reagent (Fig. 3A). S146C LacY was reconstituted into the different lipid mixes. The maleimide-PEG-biotin label is membrane impermeable and reacts with cysteine residues. The proteoliposomes were incubated with the label, then the reaction stopped by the addition of β-mercaptoethanol and the vesicles dissolved in 2% (w/v) n-octyl-β-D-glucopyranosideside (OG). The extent of labelling was compared to that when the liposomes were dissolved before labelling. The protein was separated by SDS-PAGE and stained with fluorescein-avidin. This modified avidin protein binds to the biotinylated LacY, allowing for easy in-gel quantification. The percentage of labelled protein before liposome solubilisation in OG was compared to the total amount labelled after solubilisation to give a measure of how much LacY is in the correct topology. In DOPC/DOPE mixtures S146C was found to be available to labelling, indicating high levels of correct topology, eg of ~80% in DOPC liposomes (Figs 3 and S3). Increasing the amount of charged lipid, DOPG with DOPC or DOPE present resulted in lower labelling of S146C, indicating an inverted topology as the headgroup charge increases, with <20% of the protein having the correct topology with DOPG mole fractions above 0.6 (Fig. 3). To ensure the changes in labelling were as a result of internal structural rearrangements and not from a total inversion of the protein during reconstitution, labelling was also performed at another site – S401C – on the C-terminal domain. This labelled with at least 75% efficiency at all lipid compositions measured, demonstrating only the N-domain was changing in structure.

### Folding

**Figure 4:**
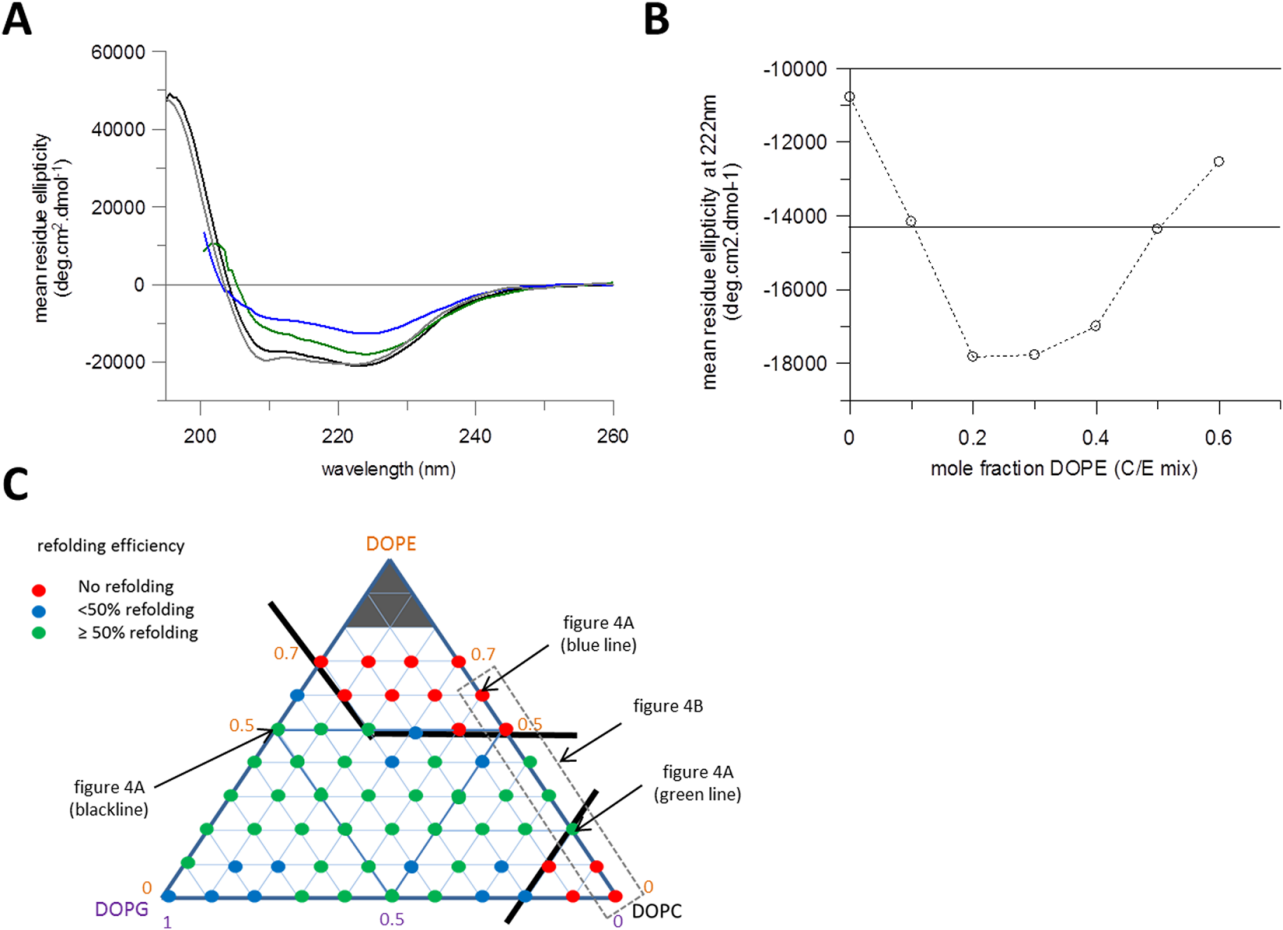
Refolding LacY into vesicles of different lipid composition. LacY was refolded from a urea-denatured state directly into lipid vesicles. (**A**) Urea denatured LacY refolded into 0.5/0.5 mole fraction DOPE/DOPG vesicles (black line). Synchrotron CD spectrum after dialysis to remove residual urea. Additionally spectra of urea-denatured LacY that has partially refolded in 0.8/0.2 DOPC/DOPE vesicles (green line), 0.4/0.6 DOPC/DOPG vesicles (blue line) and the original spectrum of folded LacY in DDM micelles (grey line) are included for comparison. (**B**) CD band intensity at 222 nm of refolded LacY in different mixes of DOPC and DOPE. DOPE concentration given as mole fraction in DOPE/DOPC vesicles. The solid line through the middle of the graph indicates the CD signal at 222 nm in the urea denatured state. (**C**) Diagrammatic representation of refolding efficiency, calculated as a percentage of added protein after a sucrose flotation to separate liposomes containing folded protein in normal or inverted topology from misfolded protein. The dots corresponding to the spectra in Fig. 4A have been highlighted and and grey dashed box shows the range of lipid mixtures from Fig. 4B.

In line with previous work on LacY and GalP^29,30^, LacY was unfolded from DDM micelles to a partially denatured state in 8M urea then refolded into liposomes. The urea denatured protein was diluted into liposomes and incubated for 30 mins. SRCD spectra were collected to assess recovery of α-helical structure after residual urea was removed by dialysis. LacY folded into 0.5/0.5 DOPE/DOPG vesicles recovered the correct secondary structure with both the intensity of the CD bands and the 210/222 ratio being equivalent to that of a reconstituted LacY sample (*e.g*. Fig. 4A). As previously predicted from studies on lipid composition effects on folding, increasing the DOPE content in DOPC vesicles reduced the amount of helical structure recovered, with a reduced intensity of 222 nm band as DOPE mole fractions increased from 0.2 to 0.6 (Fig. 4B plus example spectra in 4A). However some DOPE was required to attain maximum folding of LacY, with low yields of helix recovered with DOPE mole fractions < 0.2 and in DOPC alone. An approximation of refolding yield could also be determined by separating refolded proteoliposomes from misfolded protein by a sucrose flotation and measuring the total protein content of the liposomes, though this method does not distinguish between the normal and inverted topologies. This showed that DOPG was better than DOPC at supporting folding, with a reduction in folded LacY only occurring with high DOPE present (Fig. 4C). This is similar to findings from GalP that DOPC bilayers have lower folding yields and some DOPE being required for optimal recovery of the folded state^24^. The dependence of GalP is slightly different to LacY, with the highest yields of recovered helical structure occurring at higher DOPE mole fractions of 0.5–0.6 for GalP, but 0.2–0.4 for LacY (compare Fig. S5 ^29^ and Fig. 4B).

### Transport

**Figure 5:**
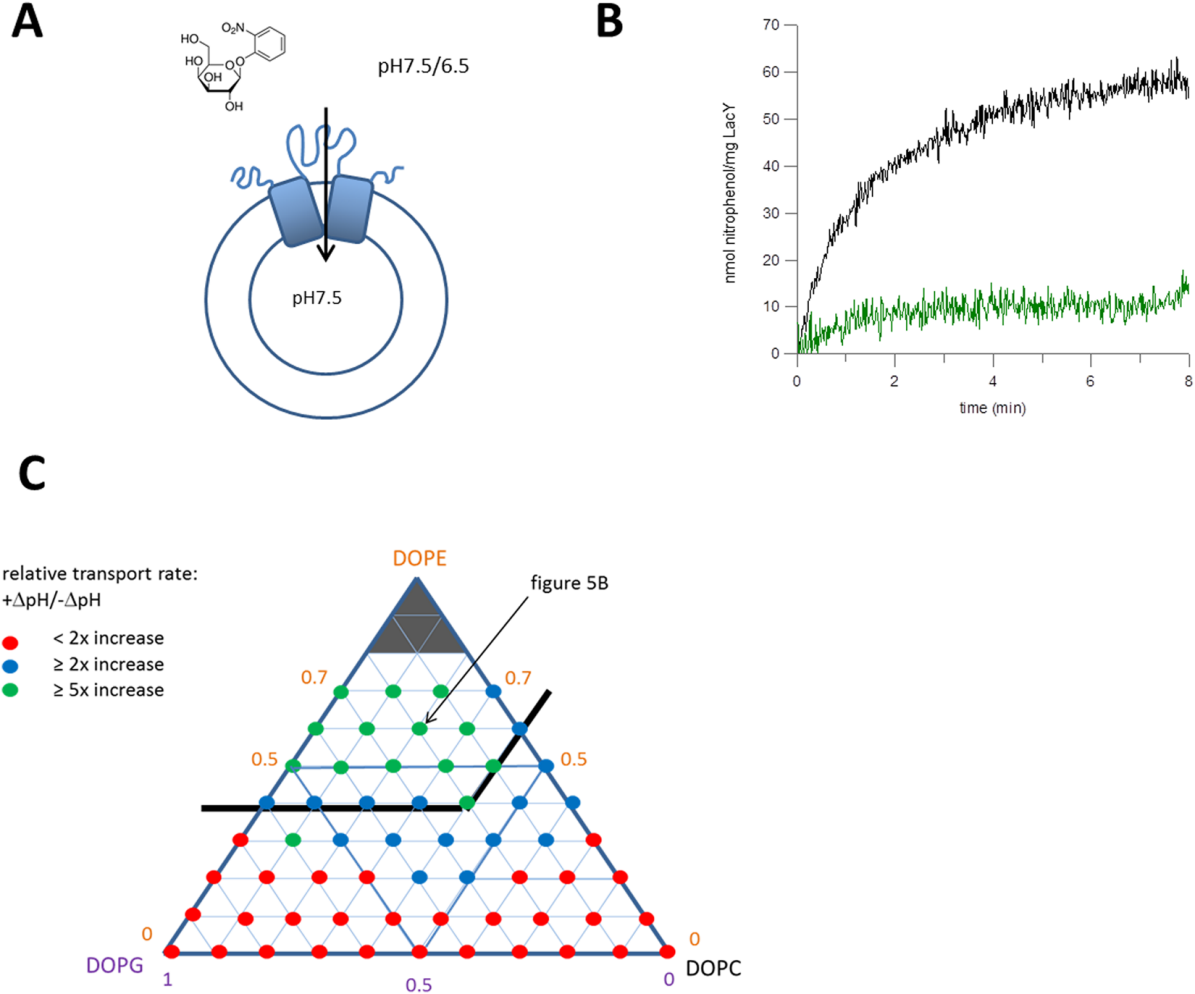
LacY transport activity in vesicles of different lipid composition. (**A**) LacY was reconstituted into vesicles with the enzyme β-galactosidase encapsulated. The substrate ο-NPG was added externally, with and without a pH gradient to measure active transport and facilitated diffusion respectively. Transported ο-NPG was digested to produce nitrophenol which was monitored by the increase in absorbance at 410 nm. (**B**) LacY reconstituted into 0.2/0.6/0.2 (mole fraction) DOPC/DOPE/DOPG vesicles showed a large increase in substrate transport in the presence of a pH gradient (black) over no gradient (green). (**C**) Diagrammatic representation of the transport activity of reconstituted LacY in vesicles composed of DOPC, DOPE and DOPG mixtures, comparing active transport to facilitated diffusion by the relative increase in initial velocity in the presence versus the absence of a pH gradient.

LacY activity is strongly influenced by bilayer composition both in cells and *in vitro* ^33,34^. LacY is a symporter, where galactoside substrates can be transported in two ways; facilitated diffusion - downhill transport – where the sugar diffuses across the membrane down its concentration gradient, or active transport – uphill transport – where a downwards pH gradient drives substrate translocation, including moving the substrate up its own concentration gradient. A colorimetric assay was used to measure transport rates of LacY reconstituted into liposomes. The enzyme β-galactosidase was incorporated into the interior of the LUVs. This enzyme cleaves the LacY substrate ο-nitrophenol galactoside (ο-NPG) to produce nitrophenol, which can be monitored by changes in absorbance at 410 nm. ο-NPG was added to the outside of the liposomes to trigger transport, with matching internal and external buffers at pH 7.5 to measure rates of downhill facilitated diffusion. Additionally, the external pH was buffered at pH 6.5 to measure active transport driven by the proton gradient. It is important that the pH gradient is maintained for the duration of these assays. This was tested and confirmed by incorporating a pH-sensitive dye inside the liposomes and monitoring any change in fluorescence, and therefore interior pH, during transport (see supplementary Figure S2). LacY reconstituted into 0.2/0.6/0.2 DOPC/DOPE/DOPG lipids showed increased transport in the presence of the pH gradient, with a 7-fold increase in the initial rate,V_o_, from 4 nmol/mg/min with no pH gradient across the proteoliposome membrane to 29 nmol/mg/min in the presence of the pH gradient. Thus protein is competent for active transport (Fig. 5B). Across the range of lipid compositions, there was little difference in rates of facilitated diffusion. However, the active transport differed considerably, with both variables being important. At least 0.4 mole fraction DOPE was required for a significant amount of uphill transport. Lipid compositions containing >0.5 mole fraction DOPG were unfavourable, which correlates with the changes in protein topology in those lipid mixes. High concentrations of DOPC also results in lower transport (Fig. 5C).

## Discussion

This study confirms the premise that lipid bilayer global properties, notably electrostatic and elastic, influence different aspects of membrane protein behaviour in different ways. Here, we sought empirical correlations with membrane protein properties over a range of lipid compositions that are known to alter electrostatic charge in the headgroup region and the lateral pressure profile of the lipid bilayer. In line with our predictions, membrane protein reconstitution, folding, topology and function exhibit different dependencies on lipid composition in DOPC/DOPE/DOPG mixtures, which in turn are likely to reflect different dependencies on headgroup charge and other lipid bilayer mechanical properties.

Reconstitution and folding of LacY in lipid vesicles exhibit similar dependencies on DOPE content with the efficiency of both processes decreasing when the DOPE mole fraction is >0.5 in DOPE/DOPC/DOPG vesicles (see Figs 1 and 4). Reconstitution involves the transfer of LacY from detergent micelles into the vesicles, with the vesicle pre-treated with detergent that partitions into the vesicles to facilitate this transfer. In spite of this pre-saturation by detergent, high levels of DOPE appear to hinder reconstitution, which involves inserting the folded protein across the bilayer. Similarly, in line with previous work^27^, high PE concentration hinders insertion across the bilayer when LacY is folded from a urea-denatured state (with no detergent present). Reconstitution and folding differ at low DOPE, as some DOPE is needed for folding but not reconstitution. Differences are also seen in the dependence on DOPG. The latter has little influence on folding from urea, however high amounts of DOPG favour reconstitution, with yields increasing from ~75% to ~90–100% with mole fractions of >0.5 DOPG in bilayers of the tri-component bilayer mixtures studied here (Fig. 1C).

Some DOPE needs to be present in the bilayer for LacY to refold efficiently from urea. This is in line with our earlier work on the related MFS protein, GalP where low PE is unfavourable for folding^29^. As observed for LacY, the folding yield of GalP increases with DOPE before reaching complete recovery of folded protein, after which at higher PE the yield decreases. The proportion of PE needed to give optimal recovery of LacY is lower than GalP: highest refolding yields occur for LacY at 0.2–0.4 mole fraction DOPE (in DOPE/DOPC bilayers) and at 0.5–0.6 mole fraction DOPE for GalP.

**Figure 6:**
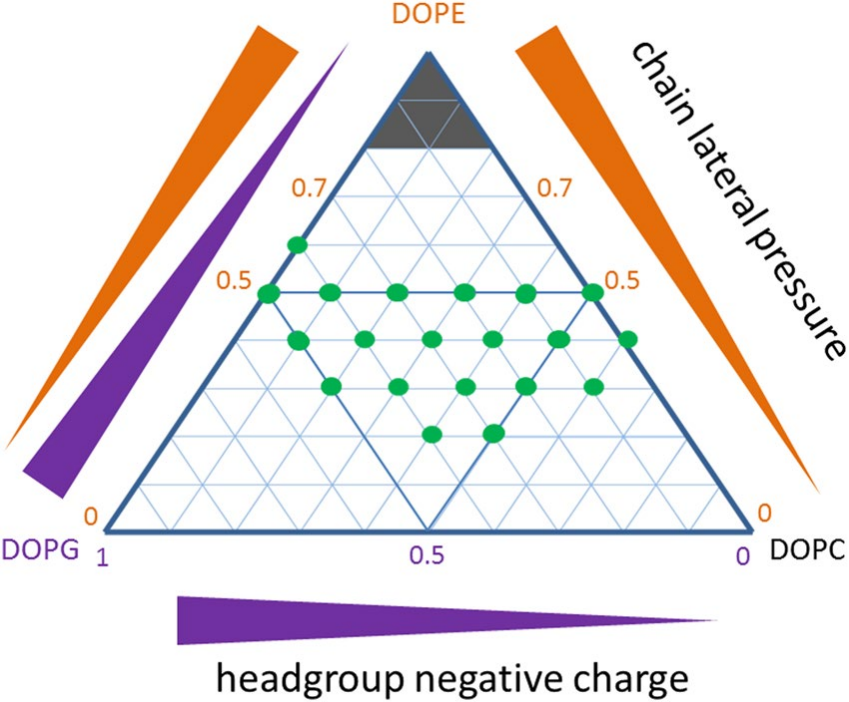
A diagrammatic representation summarising the combined data. Green dots represent the tertiary lipid compositions where all aspects of protein function are favourable.

The influence of DOPG and DOPE on several properties of reconstituted LacY was investigated. In line with the notion that an increased chain lateral pressure increases protein stability, thermal stability of LacY appeared to increase slightly with higher DOPE mole fractions. With regard to correct topology, this was observed in accordance with previous findings^33^. Topology is maintained in DOPC/DOPE mixtures, but the presence of charged DOPG above 0.4 mole fraction results in an inverted topology. Correlating with this is an absence of the ability to transport with DOPG present (as previously noted^45^), which therefore results from the inverted topology being unable to support active transport. Active, uphill, transport is the key biological function of LacY, as this allows the bacterium to utilise the proton gradient across the plasma membrane to import and concentrate galactoside substrates within the cell, and is only possible within a much narrower range of lipid compositions than transport by facilitated diffusion. The inversion in topology has previously been shown to be induced by the lipid environment and ascribed to a charge balance effect^35^. As previously noted DOPE aids active transport activity of LacY^45^. In summary, our results show that increased DOPG favours *in vitro* insertion and reconstitution yield, but is detrimental to topology and function once the protein is in the bilayer. Equally PE affects different aspects of membrane protein behaviour differently. PE is required for stability, topology and active transport as well as refolding from urea, but too high PE content limits folding/reconstitution yields. There is thus a region of lipid composition in the DOPC/DOPE/DOPG systems where all LacY properties studied are favourable, but not necessarily optimised (see Fig. 6).

Some of this variation in protein structure and function can be rationally explained by the changes in the physical properties of the bilayer. A membrane with high PE content has an increased bending rigidity, increased lateral pressure through the central hydrophobic region of the bilayer and extensive hydrogen bonding in the headgroup region^19,20,46–50^. It has been shown here to increase the stability of LacY, an effect that has also been observed with other proteins of very different structures^11,51^, and can likely be generalised more broadly to other membrane proteins. Similarly the decrease in yield of folded protein, both from reconstitution and folding, at the highest PE concentrations may arise from an increased lateral pressure in the core of the bilayer. It has previously been shown that the activation energy for inserting a helix across a bilayer increases with increased lipid chain pressure^27^. It has also been shown that the *in vitro* folding yield and kinetics of bacteriorhodopsin correlated with lipid chain lateral pressure in PC/PE mixtures^8,52^. There seems to be a requirement for intermediate concentrations of PE for efficient folding of LacY and GalP. The MFS transporters are relatively large and flexible, with two 6-helical domains, and it may be that a certain chain pressure needs to be exerted on the two domains to attain a folded state. The optimum PE concentration is somewhat different for GalP and LacY. This could be related to the N-terminal flexibility of LacY which enables the structural inversion, or just a difference in the balance of various aspects of the folding process. With further work on this, including kinetic measurements, it should be possible to isolate the differential impact of insertion and folding in the bilayer on the final folded protein yield.

Other aspects of the bilayer properties are less well understood. Although the topology inversion of LacY in high PG bilayers is relatively specific to this and some other similar proteins^12,53^, the “positive-inside” rule for membrane proteins is commonly observed, where an imbalance of positively charged amino acids on either side of the bilayer are important for directing the topology of membrane proteins^54^. It is therefore likely that large changes in bilayer charge could affect the structure and/or topology of other proteins in different ways. It can also be noted that the PC/PE and PG/PE axes are different for different properties of LacY. Although DOPG/DOPE bilayers show a similar increasing monolayer curvature with increased PE, the headgroup likely effects the lateral pressure profile in addition to the direct charge effect. More complete data on the mechanical properties of bilayers composed of these lipid mixes and especially the tertiary mixes we use here would allow further analysis of which aspects of the bilayer properties drive which protein characteristics.

It is widely accepted that lipid composition affects membrane protein function. Here, we provide a more comprehensive, systematic study giving insight into the origin of the lipid effects. Many investigations *in vitro* do not routinely check a wide range of lipid types and composition, and thus if the chosen lipid composition for study is suboptimal, this will influence results and the conclusions drawn regarding membrane protein function and other properties. Nonetheless, several studies do screen across lipids. Lipid influence has been ascribed to specific chemical interactions^55^, lipid binding pockets on the protein^4^, the influence of an annular shell of lipids^56^, hydrophobic mismatch^15^, charge effects as well as non-lamellar lipid properties. We provide evidence that global mechanical properties modulate different membrane protein properties in different ways. The fact that different membrane protein parameters are optimised by poising membrane mechanics at different points could be used exploited *in vivo* in different membranes. For example we can conjecture that the endoplasmic reticulum membrane may facilitate initial folding but a plasma membrane favours final fold and functional motions. Methods however need to be developed to probe this in cellular membranes to assess whether this is the case. In many instances membranes are likely to be poised so that all relevant protein properties are favourable. For example it has been shown that bacteria *Acholeplasma laidlawii* and *E. coli* maintain the physical state of their membranes close to a bilayer–nonbilayer phase transition, and hence if synthesis of a particular lipid is prevented, they compensate by producing another lipid with similar phase behaviour^57,58^.

Our systematic study of the influence of bilayer properties on various characteristics of LacY provides further evidence for the notion of lateral pressure influencing key membrane protein parameters. Furthermore, we show the dependence on lateral pressure across a range of bilayer headgroup charge. The predictive aspects that are emerging in this area, aid synthetic biology and nanotechnology applications of membrane proteins by beginning to lay down rules for designing efficient lipid systems that are optimised for the efficient insertion of functional proteins.

## Methods

### Materials

Lipds were purchased from Avanti Polar Lipids Inc and detergents from Anatrace. Unless otherwise stated, all other chemicals were purchased from Sigma Aldrich.

### Protein expression and purification

LacY was overexpressed and purified in *E. coli* as previously described^30^. Briefly, BL21-AI (Invitrogen) cells were transformed with the *lacy* gene cloned in the pET28 plasmid (Novagen) with a C-terminal 10-histidine tag, grown in LB media to an OD600 of 0.8 then expression was induced with 1 mM IPTG and 0.1% (w/v) arabinose and growth continued until saturation. The cells were harvested, passed through a microfluidiser (Constant Systems) and the membranes isolated by centrifugation for 30 mins at 100,000 × g. Membrane proteins were solubilised for 2 hours in 50 mM sodium phosphate, 100 mM NaCl, 10% (v/v) glycerol, 20 mM imidazole, 2 mM B-mercaptoethanol, pH 7.4 with 2% (w/v) DDM. Solubilised LacY was bound to a 1ml Histrap column (GE Healthcare) and washed with buffer containing 75 mM imidazole and 0.05% (w/v) DDM. Purified protein was eluted with 500 mM imidazole and exchanged into a final buffer of 50 mM sodium phosphate, 10% (v/v) glycerol, 1 mM B-mercaptoethanol, 0.05% DDM pH 7.4 on a 5 ml Hitrap desalting column (GE Healthcare).

### Liposome preparation and reconstitution

LacY proteoliposomes were prepared as previously described^39^. DOPC, DOPE and DOPG were dissolved at 50 mg/ml in cyclohexane, combined in the desired molar ratio, frozen in liquid nitrogen then dried overnight under vacuum. The lipid film was rehydrated to 10 mg/ml in 50 mM sodium phosphate pH 7.4 and stirred for 30 mins. Large unilamellar vesicles were made by extrusion (Avanti Mini-extruder) by passing at least 20 times through a filter of first 400 nm pore size, then 100 nm pore. To reconstitute LacY, the LUVs were presaturated with OG 1% (w/v), then protein was added to a final concentration of 0.2 mg/ml (w/v) and incubated for 30 mins. Excess detergent was removed by 3 exchanges of Bio-Beads^TM^ (BioRad) pre-equilibrated in buffer. Final protein concentration was determined by a Markwell-Lowry assay^41^ and the reconstitution efficiency calculated as a percentage of protein in the liposome compared to the initial amount of protein added.

### Circular dichroism

Circular dichroism spectra were measured at a synchrotron on beamline UV12 at ANKA, Karlsruhe Institute of Technology. The samples were loaded in a 0.2 mm pathlength cell, scans were measured from 270 to 190 nm with 1 nm intervals and an averaging time of 3 s. 4 repeat scans were averaged and a background of empty liposomes subtracted using CDTool software^59^. Thermal melts were performed on an Aviv 310 CD spectrophotometer in a 1 mm pathlength cell. The CD signal at 222 nm was measured at 2deg intervals from 25deg to 95deg. Spectra were analysed using CDTool and deconvoluted on Dichroweb using CDSSTR with the SMP180 reference set^60,61^.

### Topology

LacY with a single cysteine in the large interdomain loop was made by introducing the mutation S146C into a cysteine-free background LacY by Quikchange (Stratagene) site-directed mutagenesis. Reconstituted S146C was labelled with EZ-Link Maleimide-PEG11-Biotin (Pierce) by incubating with 100 uM of the reagent for 1 hour, either before or after solubilising the liposome with 2% (w/v) OG. Labelled protein was separated by SDS-PAGE and detected by staining the gel with 10 uM fluorescein-avidin^62^. The band densities were measured using ImageJ software^63^.

### Folding

Purified LacY was unfolded by dilution into 50 mM sodium phosphate buffer pH7.4 containing 8M urea and incubated for 5 mins. As previously shown, this results in the protein losing approximately one third of its secondary structure^30^. This substantially denatured protein was then refolded by diluting tenfold into 10 mg/ml LUVs and incubated for 30 mins. For SRCD spectra, residual urea was removed by dialysis and the proteoliposomes scanned as above in a 0.5 mm pathlength cell. Misfolded protein was separated from inserted protein by sucrose gradient flotation. The liposomes were mixed with 80% (w/v) sucrose in phosphate buffer to give a final concentration of 40% (w/v) sucrose. 400 ul was place at the bottom of a 2.2 ml tube, 1.6 ml 20% (w/v) sucrose was layered over it and a final layer of 200 ul buffer added on top. The gradient was centrifuged for 1 hour at 55,000 rpm in a TLS-55 Beckman rotor. The floated liposomes were removed and the protein concentration determined by Markwell Lowry assay.

### Transport

50 μl/ml β-galactosidase was added to the buffer during the rehydration of the lipid films and the vesicles were freeze-thawed 5 times in liquid nitrogen before extrusion to promote the incorporation of the enzyme into the liposomes. External β-galactosidase was separated by sucrose flotation. LacY was then reconstituted as above. Proteoliposomes were diluted 10-fold into phosphate buffer at either pH 7.5 or pH 6.5 and equilibrated for 2 mins. 100 μM of o-nitrophenol galactoside was added, and the production of nitrophenol in the interior of the liposomes, and therefore buffered to pH 7.5, was monitored on a Cary UV-Vis spectrophotometer measuring absorbance at 410 nm. Initial velocities were calculated and compared between samples with and without a pH gradient.

## Electronic supplementary material

Supplementary Information
