# Supplementary material for: The folding, stability and function of lactose permease differ in their dependence on bilayer lipid composition
Source: Sci Rep. 2017 Oct 12;7:13056. doi: 10.1038/s41598-017-13290-7 (PMC5638818; doi:10.1038/s41598-017-13290-7)
Supplement: Supplementary file 1 — Supplementary Information [file 41598_2017_13290_MOESM1_ESM.pdf]

Supplementary Information

The folding, stability and function of lactose permease differ in their dependence on bilayer lipid composition

Heather E Findlay and Paula J Booth*

Department of Chemistry

Kings College London

Britannia House, 7 Trinity Street

London, SE1 1DB, UK

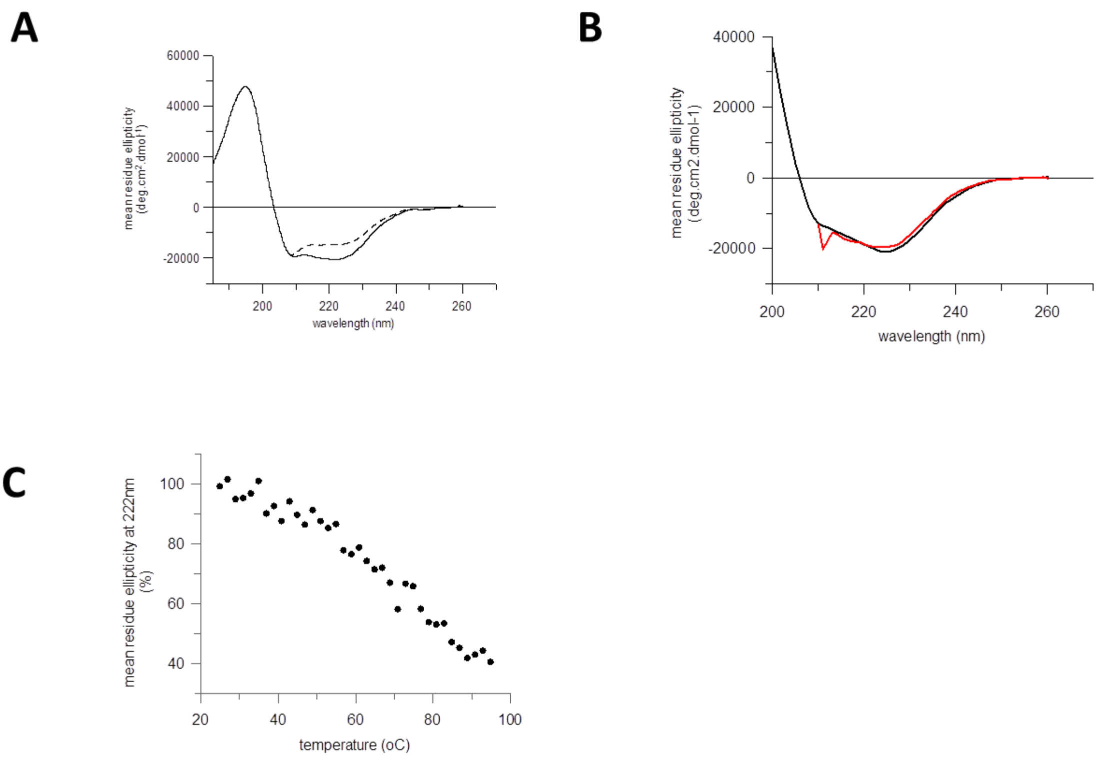

Supplementary Figure S1: A. Circular dichroism spectra of LacY in DDM detergent micelles without denaturant (solid black line) or in 8M urea (dashed black line). Approximately a third of secondary structure is lost. B. Circular dichroism spectra of LacY reconstituted into liposomes composed of 0.8/0.2 mole fraction DOPC/DOPG without denaturant (solid black line) or in 8M urea (solid red line). There is no significant change in the secondary structure of the protein. C. Thermal melt of LacY reconstituted into 0.2/0.8 mole fraction DOPC/DOPG liposomes, measured by monitoring the change in the 222nm peak of the circular dichroism spectrum across a range of temperatures. A large amount of secondary structure was lost, but the plot could not be fitted to a sigmoidal equation to give a T_m.

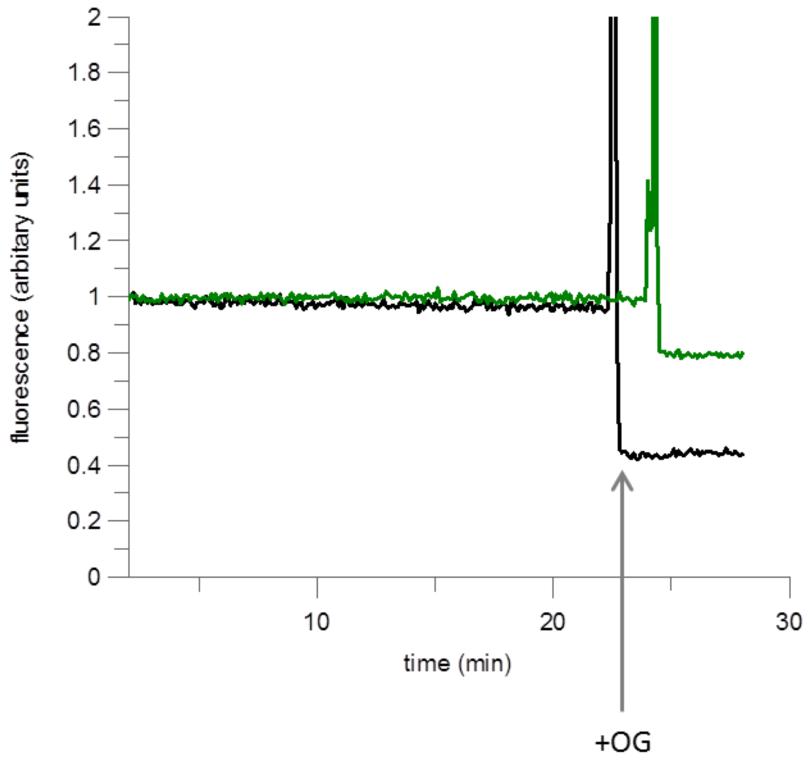

Supplementary Figure S2: Proteoliposomes composed of 0.4/0.4/0.2 DOPC/DOPE/DOPG and containing reconstituted LacY were prepared mostly by the same method as for transport assays, as described in the main text. However, instead of the enzyme β-galactosidase being incorporated inside the vesicles, the pH sensitive dye pyranine was added at a concentration of 50μM. External dye was removed by sucrose flotation. Liposomes were diluted into phosphate buffer at either pH7.5 (green line) or pH6.5 (black line), and incubated for 2mins, before the addition of substrate to initiate transport. The pH in the interior of the vesicles was monitored on a Fluoromax-4 (Horiba) fluorometer with an excitation wavelength of 454nm and an emission wavelength of 511nm. After approximately 20mins, the detergent octyl glucoside was added to a final concentration of 1% (w/v) to burst the vesicles. The liposomes buffered at pH7.5 on both sides of the bilayer showed a small drop in fluorescence, due to the dilution of the pyranine upon bursting. The liposomes with an external buffer at pH6.5 had a much larger drop due to the decrease in pH. The overlay of the two samples during the first 20mins when the vesicles are still intact demonstrates that the pH gradient is maintained during transport.

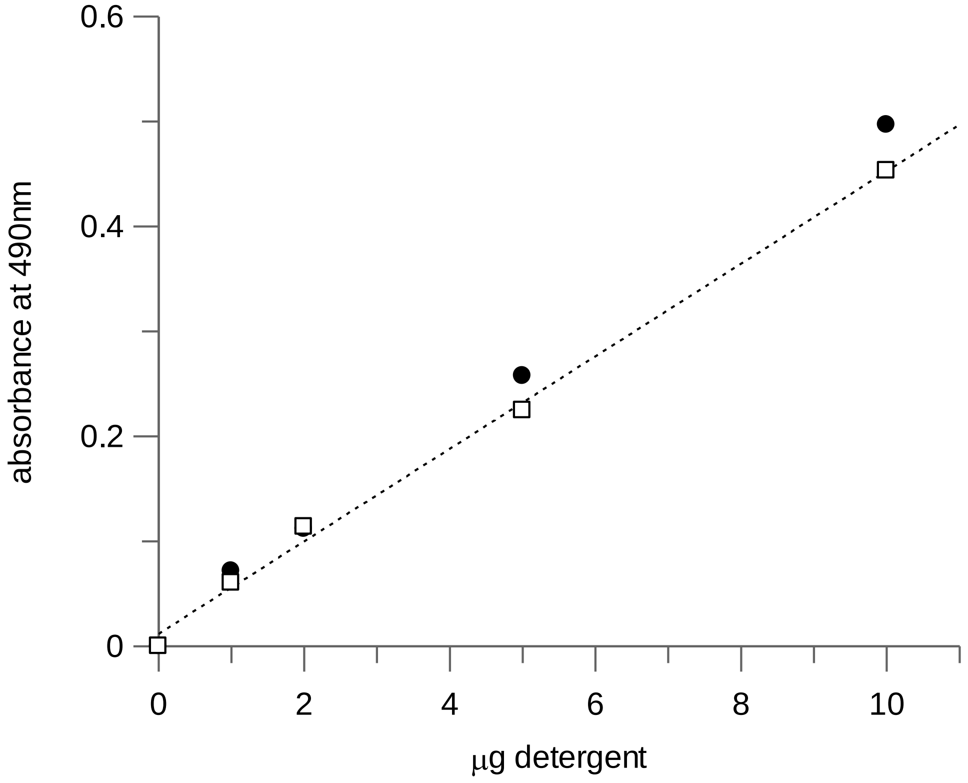

Supplementary Figure S3: The amount of residual detergent in liposomes after reconstitution or refolding can be calculated using a colorimetric assay for carbohydrate (see Taylor KACC (1995) Appl Biochem Biotech 53:207-214). Liposomes were harvested from the bulk solution by centrifugation for 90mins at 90,000 rpm in a Beckman TLA100.3 rotor, and resuspended in 50μl dH_2O. 600μl of concentrated sulphuric acid was added and the reaction allowed to proceed until it returned to room temperature. 250μl of 5% (v/v) phenol was added and after 15mins the absorbance was measured at 490nm. A standard curve was constructed from a range of concentrations of either n-octyl-β-D-glycopyranoside (open squares) or n-dodecyl-β-D-maltopyranoside (closed circles) in 50μl dH_2O which were reacted as above. There is little difference between the two detergents in this concentration range. The residual detergent can then be calculated. For example, 125μl of 0.4/0.4/0.2 PC/PE/PG proteoliposomes containing reconstituted LacY in were harvested as above and found to contain 0.5μg detergent. Assuming all the residual detergent was OG, this would be equivalent to 14μM or 1 detergent molcule per 907 lipids. However, some of this detergent will actually be DDM from the purified protein. This assay cannot distinguish between the two detergents, but as DDM has a higher molecular weight than OG it means the calculated detergent:lipid ratio is an overestimate of the molar amount of detergent present. Residual detergent present in refolded samples can be measured in the same way, though in this case only DDM is present.

DOPC	DOPE	DOPG	reconstituion efficiency (%)	+/-STD	DOPC	DOPE	DOPG	reconstituion efficiency (%)	+/-STD
1	0	0	76	7	0.6	0	0.4	96	2
0.9	0.1	0	87	5	0.5	0.1	0.4	80	7
0.8	0.2	0	87	8	0.4	0.2	0.4	80	8
0.7	0.3	0	84	4	0.3	0.3	0.4	82	3
0.6	0.4	0	81	9	0.2	0.4	0.4	73	4
0.5	0.5	0	74	13	0.1	0.5	0.4	77	3
0.4	0.6	0	59	14	0	0.6	0.4	76	5
0.3	0.7	0	56	7					
					0.5	0	0.5	84	7
0.9	0	0.1	85	4	0.4	0.1	0.5	83	5
0.8	0.1	0.1	88	4	0.3	0.2	0.5	84	7
0.7	0.2	0.1	78	5	0.2	0.3	0.5	77	7
0.6	0.3	0.1	83	3	0.1	0.4	0.5	82	2
0.5	0.4	0.1	76	10	0	0.5	0.5	80	7
0.4	0.5	0.1	77	8					
0.3	0.6	0.1	73	10	0.4	0	0.6	98	1
0.2	0.7	0.1	59	9	0.3	0.1	0.6	81	3
					0.2	0.2	0.6	86	4
0.8	0	0.2	82	2	0.1	0.3	0.6	90	4
0.7	0.1	0.2	77	7	0	0.4	0.6	91	3
0.6	0.2	0.2	76	6					
0.5	0.3	0.2	72	5	0.3	0	0.7	90	5
0.4	0.4	0.2	85	9	0.2	0.1	0.7	84	7
0.3	0.5	0.2	59	4	0.1	0.2	0.7	93	7
0.2	0.6	0.2	63	7	0	0.3	0.7	87	7
0.1	0.7	0.2	59	10					
					0.2	0	0.8	98	2
0.7	0	0.3	85	6	0.1	0.1	0.8	94	5
0.6	0.1	0.3	88	7	0	0.2	0.8	90	6
0.5	0.2	0.3	89	8					
0.4	0.3	0.3	79	4	0.1	0	0.9	91	7
0.3	0.4	0.3	84	7	0	0.1	0.9	84	3
0.2	0.5	0.3	78	5					
0.1	0.6	0.3	67	4	0	0	1	81	3
0	0.7	0.3	69	2					

Supplementary Table S1: Reconstitution. Tertiary mixtures of DOPC, DOPE and DOPG are shown as mole fractions of the total lipid present in the bilayer. The reconstitution efficiency of LacY into liposomes composed of these mixtures is shown. n≥3.

DOPC	DOPE	DOPG	correct topology (%)	+/-STD
1	0	0	83	9
0.9	0.1	0	70	4
0.8	0.2	0	78	8
0.7	0.3	0	89	8
0.6	0.4	0	69	7
0.5	0.5	0	72	5
0.4	0.6	0	68	7
0.3	0.7	0	69	7
0.9	0	0.1	91	7
0.8	0.1	0.1	90	6
0.7	0.2	0.1	70	9
0.6	0.3	0.1	87	11
0.5	0.4	0.1	84	14
0.4	0.5	0.1	58	3
0.3	0.6	0.1	91	6
0.2	0.7	0.1	82	8
0.8	0	0.2	86	5
0.7	0.1	0.2	69	6
0.6	0.2	0.2	70	7
0.5	0.3	0.2	62	5
0.4	0.4	0.2	82	8
0.3	0.5	0.2	68	4
0.2	0.6	0.2	68	8
0.1	0.7	0.2	66	5
0.7	0	0.3	73	2
0.6	0.1	0.3	67	10
0.5	0.2	0.3	68	8
0.4	0.3	0.3	87	8
0.3	0.4	0.3	49	2
0.2	0.5	0.3	60	4
0.1	0.6	0.3	69	7
0	0.7	0.3	84	7

DOPC	DOPE	DOPG	correct topology (%)	+/-STD
0.6	0	0.4	75	4
0.5	0.1	0.4	60	2
0.4	0.2	0.4	69	10
0.3	0.3	0.4	59	6
0.2	0.4	0.4	48	4
0.1	0.5	0.4	67	8
0	0.6	0.4	76	8
0.5	0	0.5	46	4
0.4	0.1	0.5	55	6
0.3	0.2	0.5	48	9
0.2	0.3	0.5	25	5
0.1	0.4	0.5	55	8
0	0.5	0.5	53	5
0.4	0	0.6	34	7
0.3	0.1	0.6	35	3
0.2	0.2	0.6	31	6
0.1	0.3	0.6	32	7
0	0.4	0.6	34	5
0.3	0	0.7	19	6
0.2	0.1	0.7	17	10
0.1	0.2	0.7	27	2
0	0.3	0.7	27	5
0.2	0	0.8	21	8
0.1	0.1	0.8	19	2
0	0.2	0.8	25	6
0.1	0	0.9	14	3
0	0.1	0.9	25	3
0	0	1	12	5

Supplementary Table S2: Topology. Tertiary mixtures of DOPC, DOPE and DOPG are shown as mole fractions of the total lipid present in the bilayer. Single cysteine mutant S146C LacY was reconstituted into liposomes composed of the mixtures and labelled with Maleimide-PEG11-Biotin before and after solubilisation with octyl glucoside. The percentage of protein that was labelled before solubilisation, indicating correct topology within the membrane, is shown. n≥3.

DOPC	DOPE	DOPG	refolded protein (%)	+/-STD
1	0	0	0	0
0.9	0.1	0	0	0
0.8	0.2	0	85	3
0.7	0.3	0	78	4
0.6	0.4	0	73	5
0.5	0.5	0	0	0
0.4	0.6	0	0	0
0.3	0.7	0	0	0
0.9	0	0.1	0	0
0.8	0.1	0.1	0	0
0.7	0.2	0.1	77	5
0.6	0.3	0.1	81	4
0.5	0.4	0.1	67	3
0.4	0.5	0.1	0	0
0.3	0.6	0.1	0	0
0.2	0.7	0.1	0	0
0.8	0	0.2	49	6
0.7	0.1	0.2	48	6
0.6	0.2	0.2	80	7
0.5	0.3	0.2	81	8
0.4	0.4	0.2	69	4
0.3	0.5	0.2	45	4
0.2	0.6	0.2	0	0
0.1	0.7	0.2	0	0
0.7	0	0.3	49	6
0.6	0.1	0.3	58	7
0.5	0.2	0.3	65	7
0.4	0.3	0.3	82	6
0.3	0.4	0.3	49	9
0.2	0.5	0.3	82	8
0.1	0.6	0.3	0	0
0	0.7	0.3	0	0

DOPC	DOPE	DOPG	refolded protein (%)	+/-STD
0.6	0	0.4	56	2
0.5	0.1	0.4	49	6
0.4	0.2	0.4	52	6
0.3	0.3	0.4	61	8
0.2	0.4	0.4	64	3
0.1	0.5	0.4	65	4
0	0.6	0.4	48	7
0.5	0	0.5	56	2
0.4	0.1	0.5	56	4
0.3	0.2	0.5	67	3
0.2	0.3	0.5	65	6
0.1	0.4	0.5	75	4
0	0.5	0.5	80	9
0.4	0	0.6	70	2
0.3	0.1	0.6	68	3
0.2	0.2	0.6	73	3
0.1	0.3	0.6	73	4
0	0.4	0.6	86	5
0.3	0	0.7	56	5
0.2	0.1	0.7	62	4
0.1	0.2	0.7	49	5
0	0.3	0.7	59	9
0.2	0	0.8	48	6
0.1	0.1	0.8	46	6
0	0.2	0.8	54	4
0.1	0	0.9	43	4
0	0.1	0.9	51	4
0	0	1	48	6

Supplementary Table S3: Folding. Tertiary mixtures of DOPC, DOPE and DOPG are shown as mole fractions of the total lipid present in the bilayer. LacY was denatured in 8M urea, before being diluted into liposomes composed of the different mixes. The percentage of the protein that was refolded into the membrane is shown. n≥3.

DOPC	DOPE	DOPG	transport (nmol/mg/min) +ΔpH	transport (nmol/mg/min) -ΔpH	+ΔpH/-ΔpH
1	0	0	3	2	1.5
0.9	0.1	0	1	1	1.0
0.8	0.2	0	3	2	1.5
0.7	0.3	0	7	6	1.2
0.6	0.4	0	5	2	2.5
0.5	0.5	0	25	6	4.2
0.4	0.6	0	14	3	4.7
0.3	0.7	0	21	6	3.5
0.9	0	0.1	2	2	1.0
0.8	0.1	0.1	2	1	2.0
0.7	0.2	0.1	4	3	1.3
0.6	0.3	0.1	29	6	4.8
0.5	0.4	0.1	4	1	4.0
0.4	0.5	0.1	40	6	6.7
0.3	0.6	0.1	15	2	7.5
0.2	0.7	0.1	32	4	8.0
0.8	0	0.2	6	5	1.2
0.7	0.1	0.2	4	6	0.7
0.6	0.2	0.2	3	3	1.0
0.5	0.3	0.2	16	5	3.2
0.4	0.4	0.2	33	4	8.3
0.3	0.5	0.2	45	6	7.5
0.2	0.6	0.2	29	4	7.3
0.1	0.7	0.2	45	7	6.4
0.7	0	0.3	5	4	1.3
0.6	0.1	0.3	1	1	1.0
0.5	0.2	0.3	7	2	3.5
0.4	0.3	0.3	26	6	4.3
0.3	0.4	0.3	19	4	4.8
0.2	0.5	0.3	18	2	9.0
0.1	0.6	0.3	45	4	11.3
0	0.7	0.3	38	3	12.7

DOPC	DOPE	DOPG	transport (nmol/mg/min) +ΔpH	transport (nmol/mg/min) -ΔpH	+ΔpH/-ΔpH
0.6	0	0.4	3	2	1.5
0.5	0.1	0.4	2	1	2.0
0.4	0.2	0.4	11	5	2.2
0.3	0.3	0.4	11	5	2.2
0.2	0.4	0.4	14	3	4.7
0.1	0.5	0.4	17	2	8.5
0	0.6	0.4	31	4	7.8
0.5	0	0.5	5	3	1.7
0.4	0.1	0.5	6	6	1.0
0.3	0.2	0.5	2	1	2.0
0.2	0.3	0.5	13	4	3.3
0.1	0.4	0.5	9	2	4.5
0	0.5	0.5	29	5	5.8
0.4	0	0.6	7	5	1.4
0.3	0.1	0.6	1	1	1.0
0.2	0.2	0.6	4	5	0.8
0.1	0.3	0.6	16	3	5.3
0	0.4	0.6	21	6	3.5
0.3	0	0.7	7	5	1.4
0.2	0.1	0.7	6	4	1.5
0.1	0.2	0.7	1	1	1.0
0	0.3	0.7	4	3	1.3
0.2	0	0.8	2	2	1.0
0.1	0.1	0.8	5	3	1.7
0	0.2	0.8	4	5	0.8
0.1	0	0.9	2	2	1.0
0	0.1	0.9	2	1	2.0
0	0	1	5	4	1.3

Supplementary Table S4: Transport activity. Tertiary mixtures of DOPC, DOPE and DOPG are shown as mole fractions of the total lipid present in the bilayer. Liposomes were prepared with β-galactosidase enzyme incorporated inside and LacY reconstituted into the membrane. The initial transport rate of the substrate o-nitrophenol galactoside (measured by the production of the cleaved product nitrophenol) in the presence and absence of a pH gradient was measured, and the ratio of the two calculated as a measure of active transport. n≥3.
